# *LAMB2* novel variant c.2885‐9 C>A affects RNA splicing in a minigene assay

**DOI:** 10.1002/mgg3.1704

**Published:** 2021-05-13

**Authors:** Xiaoyuan Wang, Huijie Xiao, Baige Su, Yali Ren, Jie Ding, Fang Wang

**Affiliations:** ^1^ Department of Pediatrics Peking University First Hospital Beijing China; ^2^ Laboratory of Electron Microscopy Ultrastructural Pathology Center Peking University First Hospital Beijing China

**Keywords:** intronic variant, *LAMB2*, Pierson syndrome, splicing

## Abstract

**Background:**

Both Pierson syndrome (PS) and isolated nephrotic syndrome can be caused by *LAMB2* biallelic pathogenic variants. Only 15 causative splicing variants in the *LAMB2* gene have been reported. However, the pathogenicity of most of these variants has not been verified, which may lead to incorrect interpretation of the functional consequence of these variants.

**Methods:**

Using high‐throughput DNA sequencing and Sanger sequencing, we detected variants in a female with clinically suspected PS. A minigene splicing assay was performed to assess the effect of *LAMB2* intron 20 c.2885‐9C>A on RNA splicing. We also performed the immunohistochemical analysis of laminin beta‐2 in kidney tissues.

**Results:**

Two novel *LAMB2* heteroallelic variants were found: a paternally inherited variant c.2885‐9C>A in intron 20 and a maternally inherited variant c. 3658C>T (p. (Gln1220Ter)). In vitro minigene assay showed that the variant c.2885‐9C>A caused erroneous integration of a 7 bp sequence into intron 20. Immunohistochemical analysis revealed the absence of glomerular expression of laminin beta‐2, the protein encoded by *LAMB2*.

**Conclusion:**

We demonstrated the impact of a novel *LAMB2* intronic variant on RNA splicing using the minigene assay firstly. Our results extend the mutational spectrum of *LAMB2*.

## Graphical abstract

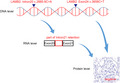

## 1 INTRODUCTION

The human *LAMB2* gene on chromosome 3p21 encodes laminin subunit beta‐2, which is expressed in the basement membrane of the glomerulus, intraocular muscles and muscles at the neuromuscular junctions. Recessive mutations in *LAMB2* can cause either Pierson syndrome (PS, OMIM: #609049) or isolated nephrotic syndrome with onset in the first year of life (OMIM: #614199) (Cil & Perwad, 2018; Zenker et al., 2004). PS is defined by abnormalities affecting the kidney, eye and the nervous system (Matejas et al., 2010; Pierson et al., 1963). Renal disease is primarily characterized by congenital nephrotic syndrome with diffuse mesangial sclerosis and early‐onset end‐stage renal disease (ESRD). Only a few patients with onset of nephrotic proteinuria and ESRD beyond 1 year of age and additional glomerular histological lesions such as focal segmental glomerulosclerosis have been reported (Cil et al., 2015). Ocular anomalies are typically described as microcoria. Among the neurological deficiencies observed in patients with PS, neurodevelopmental delay is the most frequently observed form.

Although a clinical diagnosis of PS can be made based on the combination of congenital or infantile nephrotic syndrome and microcoria, genetic testing is still needed to confirm the diagnosis. Molecular genetic testing of *LAMB2* is also critical for individuals with multiple phenotypes beyond that of classic PS. The Human Gene Mutation Database (HGMD; http://www.hgmd.cf.ac.uk/ac/index.php; last accessed Jan 31, 2021) has documented more than 130 *LAMB2* variants, and the vast majority of these variants are single‐base substitutions. Among the single‐base changes, only 15 causative splicing variants have been identified, including 12 single‐base changes where a shift in the canonical splice sites and three single‐base changes where a shift closes to the canonical sites had been identified (Houdayer et al., 2008; Minamikawa et al., 2020; Nagano et al., 2020). These findings indicate that *LAMB2* pathogenic splicing variants are uncommon.

With advances in genomic DNA sequencing technology, it is easy to analyse the entire sequence of the *LAMB2* gene. However, the molecular diagnosis of *LAMB2*‐related diseases remains challenging because of the difficulty in correctly assessing the impact of *LAMB2* variants of unknown significance. Functional analyses of these variants are, thus, required to determine the pathogenicity.

Here, we present a patient with clinically suspected PS who was compound heterozygous for two novel *LAMB2* variants: one was a non‐sense variant and the other was an intronic variant occurring in the intronic position outside the canonical acceptor site. We demonstrated the pathogenicity of the intronic variant using an in vitro minigene assay. The patient lacked laminin beta‐2 expression in the glomerular basement membrane.

## 2 MATERIALS AND METHODS

### 2.1 Ethical compliance

Written informed consent was obtained from the parents. Ethical committee approval was obtained from the Ethical Committee of Peking University First Hospital.

### 2.2 Clinical description of proband

**FIGURE 1:**
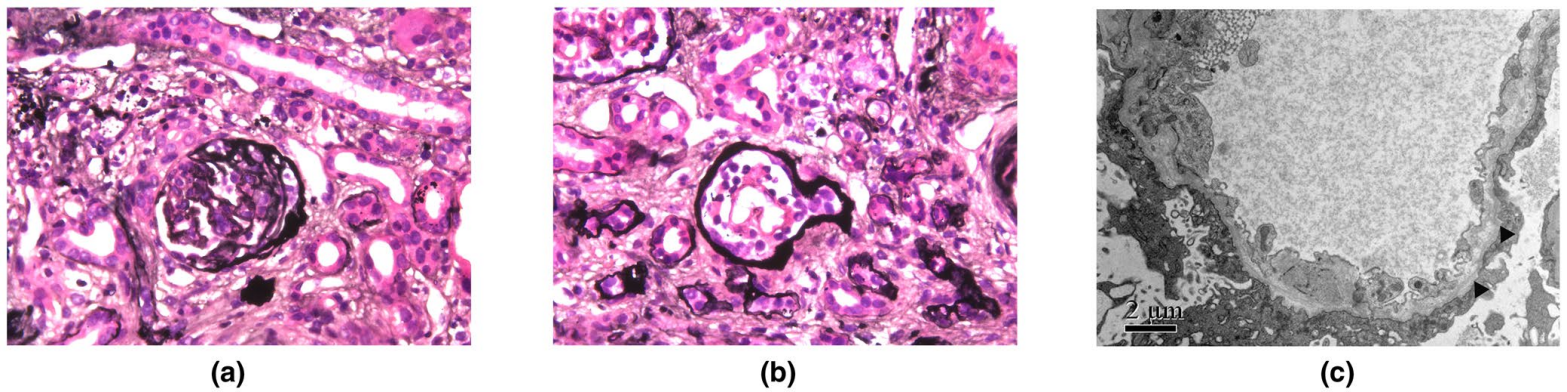
Renal biopsy findings of the proband. (a) and (b) (PASM, 400×). Light microscopy showed a sclerotic glomerulus (a) and an immature glomerulus with tubular atrophy (b). (c) Electron microscopy demonstrated a moth‐eaten appearance of the glomerular basement membrane with diffuse foot process effacement (black triangle.10000×)

A 16‐month‐old female of Han ethnicity was referred to our hospital because of proteinuria and haematuria. She initially visited the local hospital for oedema in eyelids and both legs and decreased urinary frequency for less than 7 days after a preceding upper respiratory tract infection. Over the 10 days prior to admission, the patient was diagnosed as having nephrotic syndrome from findings of massive proteinuria (dipstick 3+ on two consecutive days), hypoalbuminemia (24.7 g/L) and hypercholesterolemia (6.05 mmol/L). Urinalysis showed microscopic haematuria (40–50 red blood cells/high power field). She was born after a full term and uneventful pregnancy as the only child of a non‐consanguineous healthy couple and had a normal birth weight. Her past medical history and family history were unremarkable. She showed normal development, with no abnormal neurological signs. Physical examination on admission showed she had short stature with a height of 72 cm (<3rd percentile of the reference for Chinese children) and a weight of 9.15 kg (approximately 10–25th percentile of the reference for Chinese children), regrettably, which did not draw attention of her parents. Other physical findings were unremarkable. The features of nephrotic syndrome were confirmed by repeated tests, serum total protein 43.1 g/L, albumin 25.1 g/L, total cholesterol 6.65 mmol/L and non‐selective massive proteinuria (random urinary protein/creatinine ratio: 42.95 g/g). The proportions of urinary albumin, macromolecular‐weight protein and low‐molecular‐weight protein were 59.3%, 33.3% and 7.4%, respectively, and the patient showed microscopic haematuria (20–30 red blood cells/high power field). Serum creatinine was 55.7 µmol/L, and the evaluated glomerular filtration rate using Schwartz formula (Schwartz et al., 1987) was 51.7 ml/min/1.73 m^2^. Serum screening tests for IgG, IgM, IgA, C3, C4, antinuclear antibody, double‐stranded DNA, viral infections (including hepatitis B virus, hepatitis C virus and human immunodeficiency virus) and syphilis were normal. Renal ultrasound showed two normal‐size kidneys (7 cm in length) with dedifferentiation and hyper‐echogenicity. The patient's bone age was 2 years. Bilateral non‐reactive microcoria was detected by ophthalmological examination. A histological diagnosis of focal segmental glomerulosclerosis with tubulointerstitial injury was made based on the features of renal biopsy on the fourth week after onset (Figure 1). The patient's parents did not accept angiotensin‐converting enzyme inhibitor or angiotensin receptor blocker therapy. The patient's serum creatinine rose to 220 µmol/L at 20 months of age; at 27 months of age, she developed ESRD. However, her parents refused dialysis. The patient's final outcome information was absent because of failing to be followed up over the telephone.

### 2.3 Targeted next‐generation sequencing and data analyses

Genomic DNA was extracted from peripheral blood lymphocytes of the patient and her parents using the QIAamp DNA Blood Mini Kit (A1120, Qiagen). The quality of DNA was assessed by NanoDrop (Thermo). Targeted next‐generation sequencing, variant calling and variant annotation were performed as described previously (Wang et al., 2017). In brief, the patient was tested using a panel containing 28 known nephrotic syndrome genes. Sequence reads were mapped to the human reference genome (hg19) using BWA software. Less reliable variant calls and common variants were filtered out. The prediction of deleteriousness of unpublished variants was performed using multiple in silico tools including SIFT, PolyPhen 2, Mutation Taster, fathmm‐MKL_coding, CADD, GERP++_RS and SiPhy, and the splicing effect was predicted using Human Splicing Finder, MaxEntScan and NetGene2 Server. The pathogenicity of variants was interpreted according to American College of Medical Genetics and Genomics guidelines (Richards et al., 2015).

### 2.4 Sanger sequencing

The *LAMB2* variants that were identified with next‐generation sequencing (NM_002292.3: c.2885‐9C>A and c.3658C>T) were confirmed in the proband and her parents by PCR and Sanger sequencing as previously described (Zhao et al., 2010). The following variant‐specific primers were designed through Primer3 online: Intron 20 primers, F: 5′‐TGAAAGGTGAGACTGGAGCA‐3′ and R: 5′‐GAACCCCAATTCAGCCATGC‐3′; and Exon 24 primers, F: 5′‐GTTGCAGTGCCATGGTGAG‐3′ and R: 5′‐CCAATTTCACGCCTGCAATG‐3′.

### 2.5 In vitro splicing assays

A minigene splicing assay was performed to assess the effect of *LAMB2* intron 20 c.2885‐9C>A on RNA splicing. A 3413 bp fragment of *LAMB2* gene, including exons 18–27 and introns 18–26, was used to construct minigenes. The wild‐type and mutant sequences were synthesized and inserted into the pcDNA3.1 (+) splicing vector by GenScript (Piscataway, NJ, USA) using *NheI*/*NotI* restriction sites. After plasmid amplification in DH5α *Escherichia coli* and plasmid extraction (OMEGA), the sequences and correct orientations of all constructs were validated using pCAS2.1‐specific primers by Sanger sequencing.

HEK293T cells were grown to 80% confluence in 6‐well plates and transfected in duplicate with no vector, empty vector, the wild‐type vector or the mutant vector (1 µg total plasmid DNA per sample) using Lipofectamine™ 2000 CD Transfection Reagent (12566014, Invitrogen). Cells were harvested 24 h later, RNA was extracted from each sample using TRIzol reagent (15596018, Life Technologies), and reverse transcription was performed with the Revert Aid First Strand cDNA Synthesis Kit (K1621, Thermo Scientific) according to the manufacturer's instructions. A 626 bp fragment of *LAMB2* cDNA including exons 19–22 was amplified by PCR using a pair of primers (F: 5′‐CTGTGAAAGGTGCATTGCTGG‐3′, R 5′‐GCCCTGTGAACTCGTTGCAG‐3′) and Premix Ex Taq™ Hot Start Version (12344040, Thermo Scientific). The optimized amplification parameters were 30 cycles of 94°C for 30 s, 55°C for 1 min and 72°C for 30 s. The PCR amplification products were checked by 3% agarose gel electrophoresis and sequenced on an ABI 3730XL (SinoGenoMax Company Limited).

### 2.6 Immunohistochemical analysis of laminin beta‐2 in kidney tissues

Paraffin‐embedded renal tissues were cut into 5‐µm‐thick slices. The sections were incubated with the primary antibody rabbit anti‐human laminin beta‐2 (1:50; 223869, Life Science) at 4°C overnight and then incubated with secondary antibody conjugated (PV‐9000, Zhongshan Golden Bridge Biotechnology, China) to horseradish peroxidase. After counterstaining with haematoxylin, the sections were photographed under a BX53 microscope (Olympus).

## 3 RESULTS

**FIGURE 2:**
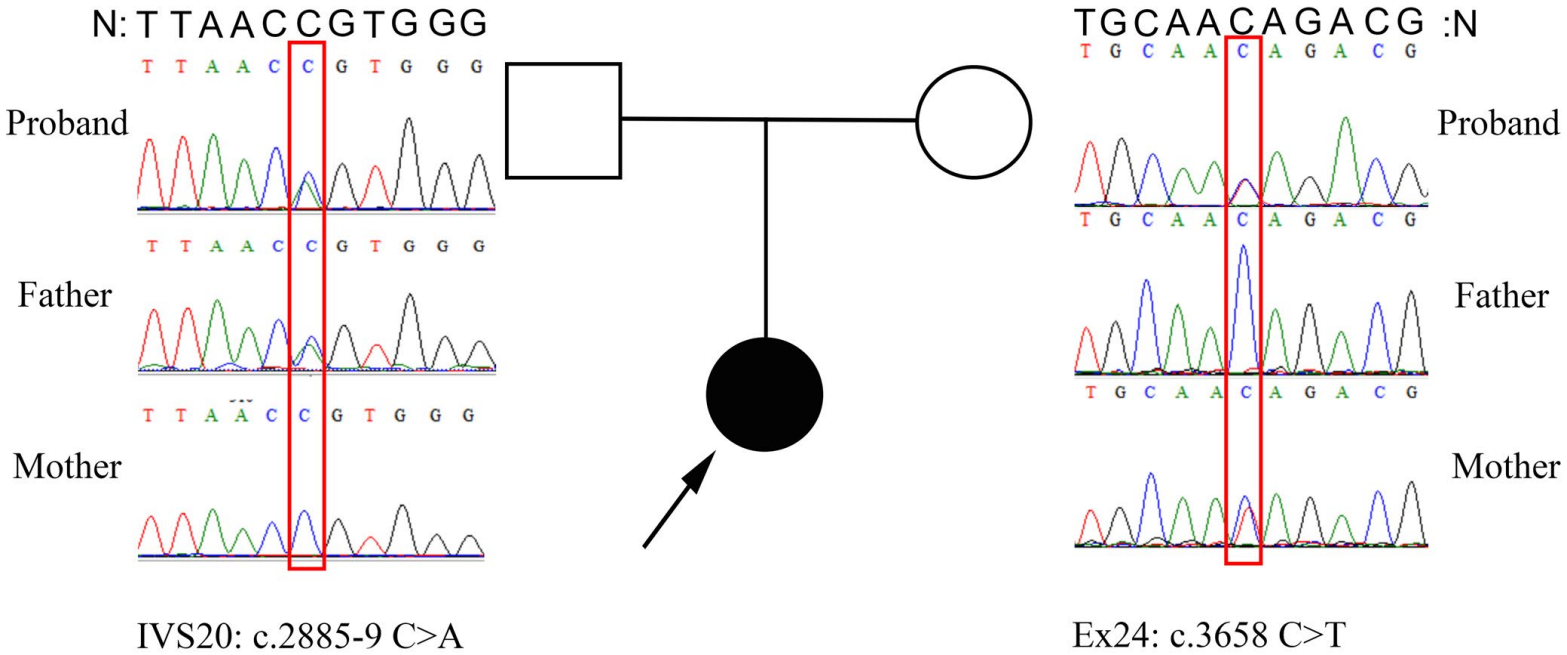
Sequencing of PCR‐amplified products of *LAMB2* intron 20 and exon 24 from the proband and her parents. N: normal sequence. Red rectangles indicate the variations; the filled black circle indicates the individual with clinically suspected Pierson syndrome; and the arrow indicates the proband

Targeted next‐generation sequencing in the proband revealed two heterozygous candidate variants in *LAMB2*: c.2885‐9C>A in intron 20 and c.3658C>T leading to premature stop codon formation p. (Gln1220Ter) in exon 24. Sanger sequencing revealed that the two variants were found on different chromosomes for the proband and her parents (Figure 2).

The frequencies of c. 3658C>T (p. Gln1220Ter) and c.2885‐9C>A variants in *LAMB2* in the control databases and disease databases, and multiple in silico predictions were indicated in Table 1. According to American College of Medical Genetics and Genomics guidelines (Richards et al., 2015), the exonic variant was classified as pathogenic (PVS1 + PM2 + PP3 + PP4), whereas the intronic variant was unclassified (PM2 + PM3 + PP4).

**TABLE 1:** Assessing the pathogenicity of two LAMB2 variants identified in the case

Sequence variant	NCBI dbSNP	1,000 Genomes Project	ESP6500	ExAC	gnomAD	HGMD	ClinVar	Mutation taster	fathmm‐MKL_coding	CADD	GERP++_RS	SiPhy	HSF	NetGene2	MaxEntScan
c.3658C>T, p. Gln1220Ter	—	—	—	—	—	—	—	1 (damaging)	0.983 (damaging)	37 (deleterious)	5.84 (deleterious)	20.128 (deleterious)	A high probability of disrupting gene splicing	No change	No change
c.2885‐9C>A	—	—	—	—	—	—	—	NA	NA	NA	NA	NA	A high probability of disrupting gene splicing	A high probability of disrupting gene splicing	A high probability of disrupting gene splicing

The SIFT and PolyPhen 2 scores for the exonic variant were not available.

Abbreviation: NA, not applicable.

**FIGURE 3:**
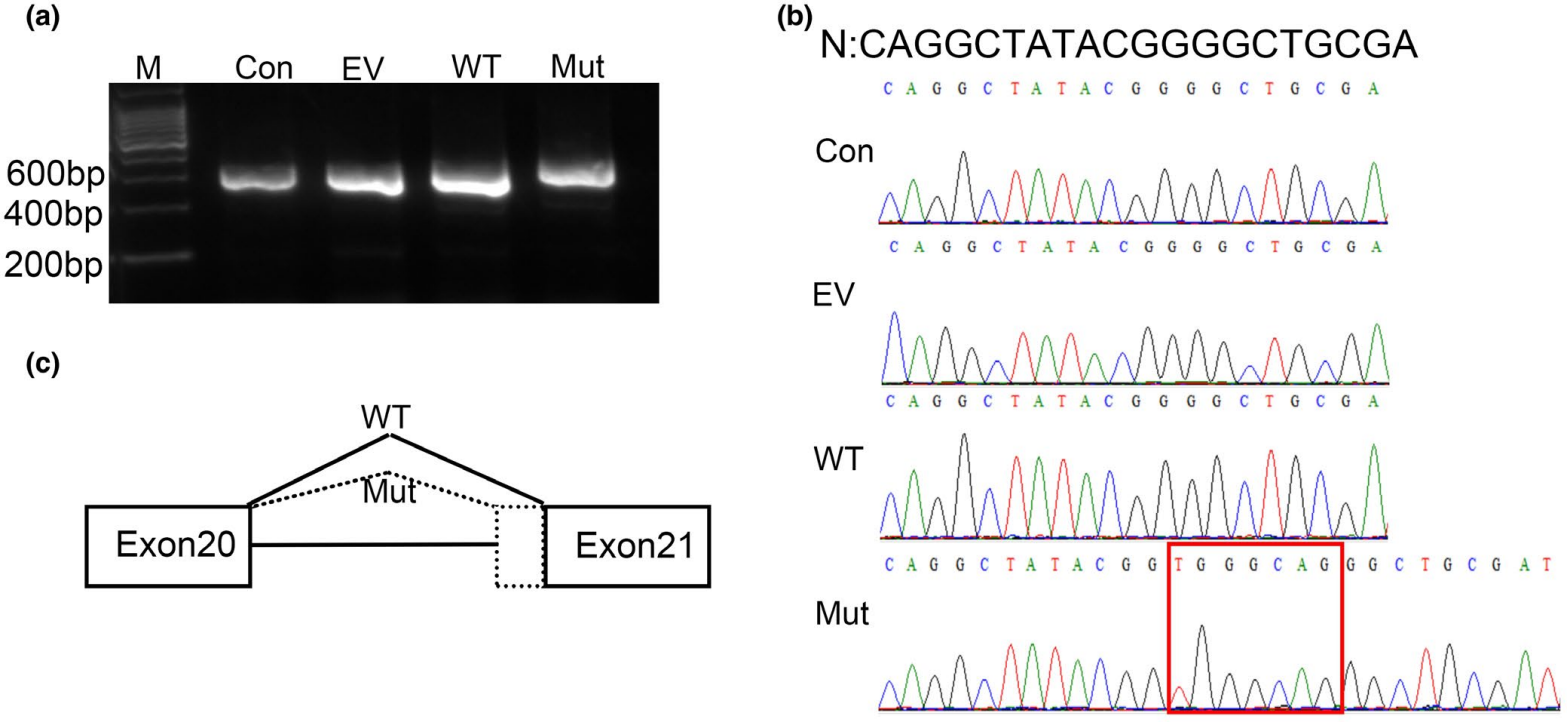
Transcript analyses of *LAMB2* variant c.2885‐9 C>A. (a) Agarose gel images of RT‐PCR products for HEK293T cells. Con: control clone; EV: empty vector clone; WT: wild‐type clone; Mut: mutation clone. M: DNA molecular mass marker. Compared with the control, empty vector and wild‐type clones, the mutation clone had a single subtly larger sized RT‐PCR product. (b) Sequencing of the RT‐PCR products from each clone. N, normal sequence. The red rectangle indicates the retention of a 7 bp sequence in intron 20 between exon 20 and exon 21. C. Schematic representation of the aberrant *LAMB2* cDNA caused by the variant c.2885‐9C>A

In silico splicing analysis using HSF, NetGene2 and MaxEntScan showed that the variant c.2885‐9C>A in *LAMB2* had a high probability of disrupting gene splicing. Therefore, molecular RNA analysis of this variant was performed. Agarose gel electrophoresis revealed that the variant c.2885‐9C>A led to a subtly larger *LAMB2* mRNA transcript than the wild‐type clone (Figure 3a). Sanger sequencing of the RT‐PCR products revealed the retention of a 7 bp sequence in intron 20 between exon 20 and exon 21 (r.2884_r.2885ins [2885‐7_2885‐1], p. Gly962Valfs*7) exclusively in the variant c.2885‐9C>A clone (Figure 3b). According to American College of Medical Genetics and Genomics guidelines (Richards et al., 2015), this variant was classed as pathogenic (PS3 + PM2 + PM3 + PM4 + PP4).

**FIGURE 4:**
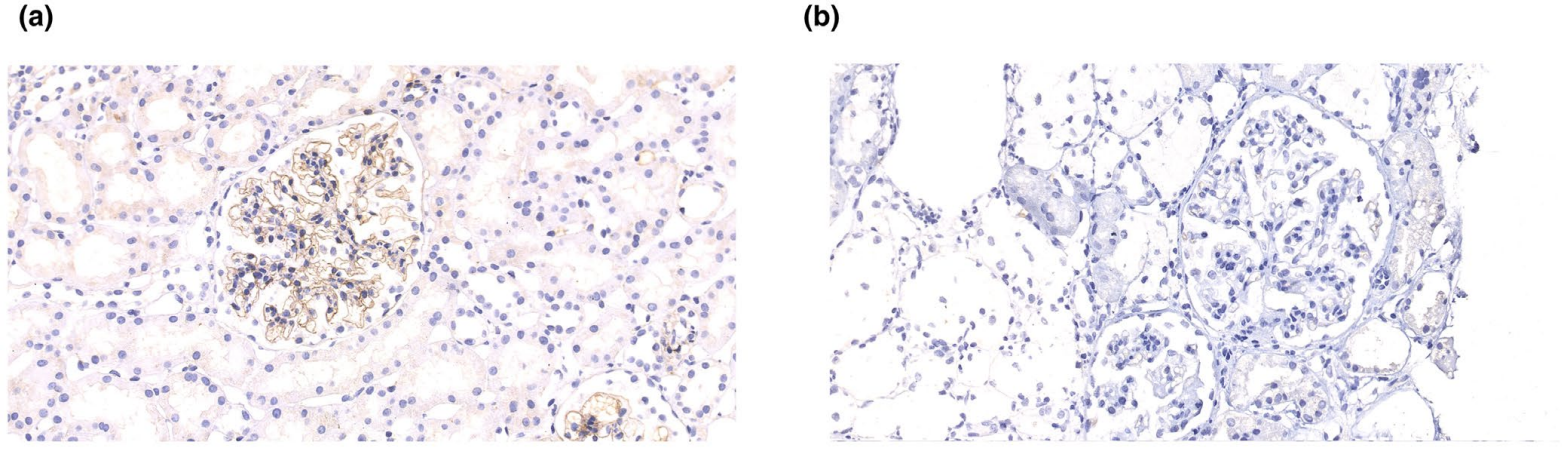
Immunohistochemical analysis of laminin beta‐2 expression in kidney specimens (200×). The linear expression pattern of laminin beta‐2 in the glomerular basement membrane was observed in an age‐matched healthy control (a. brown color), whereas there was no binding of anti‐ laminin beta‐2 antibody to the glomerular basement membrane from the proband (b)

As shown in Figure 4, the proband was found to have negative staining for laminin β2 in the glomerular basement membrane.

## 4 DISCUSSION

PS was highly suspected in the proband with nephrotic syndrome, rapidly progressive renal failure and bilateral non‐reactive microcoria. However, the clinical findings including onset age of 16 months and pathologic feature of focal segmental glomerulosclerosis were atypical, and thus, molecular genetic testing was required for a definite diagnosis. Two heterozygous novel *LAMB2* variants were detected in the patient using high‐throughput DNA sequencing and Sanger sequencing. Although the pathogenicity of the non‐sense variant was obvious, the pathogenicity of the intronic variant c.2885‐9C>A was unknown. In vitro minigene assay revealed that the intronic variant resulted in aberrant splicing, which would lead to a truncated laminin beta‐2 protein. Furthermore, complete lack of glomerular laminin beta‐2 expression was observed in kidney sections of the patient. From these results, the patient in this study was diagnosed with PS. It is worth noting that the onset of nephrotic syndrome beyond 1 year of age in the patient could not be explained by *LAMB2* biallelic truncating variants and a lack of laminin beta‐2, as Choi et al. have reported previously (Choi et al., 2008). This phenomenon might be related to some compensatory role of other laminin beta isoforms for laminin beta‐2 deficiency (Noakes et al., 1995).

Previous studies have reported PS patients with different presentations compared with the current case. Abnormal lens and obvious motor delay were observed in a PS patient with focal segmental glomerulosclerosis and microcoria, who carried homozygous *LAMB2* missense variants (p. Arg246Trp) (Bredrup et al., 2008). Severe hypotonia was reported in a PS patient with focal segmental glomerulosclerosis and microcoria, who had homozygous *LAMB2* deletion variants (p. Cys682Phefs∗13) (Goldschmidt et al., 2017). Ocular abnormalities other than microcoria were found in two patients with PS with onset of nephrotic proteinuria beyond 1 year of age and focal segmental glomerulosclerosis who had homozygous or compound heterozygous *LAMB2* truncated variants (Matejas et al., 2006, 2010). Reasons for these extrarenal differences remain to be elucidated.

Sequence changes that affect conserved nucleotides at the splice acceptor and donor sites are assumed to cause aberrant RNA splicing and considered to be pathogenic (Richards et al., 2015). However, it is difficult to correctly assess the consequences of variants located in the intronic position outside the canonical donor or acceptor sites. RNA analysis of such variants is a powerful tool to determine the outcome. For example, Minamikawa et al. (2020) found that the *LAMB2* variant c.3797 +5G>A affected RNA splicing by molecular analysis of RNA extracted from the patient's leukocytes and urine‐derived cells. A number of studies have shown that the in vitro minigene assay is an attractive alternative method to assess the impact of intronic variants on splicing, because the results of these assays are in good agreement with those of studies using patient‐derived RNA (Bonnet et al., 2008; Maselli et al., 2009; Matejas et al., 2011). Given the importance of molecular RNA analysis of *LAMB2* intronic variant c.2885‐9C>A and the unavailability of specimens from our patient such as fresh blood, urine or skin tissue (Maselli et al., 2009; Matejas et al., 2011), we used the minigene assay to analyse the consequence of the variant. As described above, the variant activated a cryptic splice site. Because of the difficulty of obtaining appropriate tissues from patients with *LAMB2*‐related diseases, the pathogenicity of most reported *LAMB2* splice site variants have not been verified by RNA studies. Our study demonstrated that the minigene assay is a useful strategy for solving this dilemma.

Several reports showed that patients with biallelic putative *LAMB2* truncating variants exhibited a complete lack of renal laminin beta‐2 expression, whereas those with presumed non‐truncating *LAMB2* variants on at least one allele showed residual or even normal glomerular expression of laminin beta‐2 (Maselli et al., 2009; Tahoun et al., 2020; Zenker et al., 2004). A similar phenomenon was observed in our patient with biallelic *LAMB2* truncating variants. Because the primary antibody used in our study is targeted for a peptide corresponding to amino acids 80–130 of human laminin beta‐2, we speculated that laminin beta‐2 deficiency might result from accelerate degradation of abnormal protein due to decreased stability (Zenker et al., 2004) or mRNA transcribed from *LAMB2* gene due to non‐sense‐mediated mRNA decay (Sawyer et al., 2010). However, two cases that were not consistent with the above‐mentioned correlation pattern were reported (Minamikawa et al., 2020). One case harbouring a compound heterozygous cryptic splice site variant in *LAMB2* along with another frameshift variant in *LAMB2* demonstrated normal glomerular staining of laminin beta‐2. Another case harbouring a homozygous presumed *LAMB2* missense variant displayed no glomerular laminin beta‐2 immunoreactivity. These data suggest that predicting the consequence of *LAMB2* variants on laminin beta‐2 production may be complicated, and other possible modifiers other than *LAMB2* genotypes may also affect protein expression (Matejas et al., 2010).

In summary, we applied the in vitro minigene assay to verify the pathogenicity of a novel *LAMB2* intronic variant, thereby extending this gene mutational spectrum and highlighting the importance of functional assessment of intronic sequence variants in *LAMB2* in diagnosis of PS.

## CONFLICT OF INTEREST

The authors declare that they have no conflict of interest.

## Data Availability

All data needed to evaluate the conclusions are present in the paper.

## References

[mgg31704-bib-0001] Bonnet, C., Krieger, S., Vezain, M., Rousselin, A., Tournier, I., Martins, A., Berthet, P., Chevrier, A., Dugast, C., Layet, V., Rossi, A., Lidereau, R., Frebourg, T., Hardouin, A., & Tosi, M. (2008). Screening BRCA1 and BRCA2 unclassified variants for splicing mutations using reverse transcription PCR on patient RNA and an ex vivo assay based on a splicing reporter minigene. Journal of Medical Genetics, 45(7), 438–446. 10.1136/jmg.2007.056895 18424508

[mgg31704-bib-0002] Bredrup, C., Matejas, V., Barrow, M., Bláhová, K., Bockenhauer, D., Fowler, D. J., Gregson, R. M., Maruniak‐Chudek, I., Medeira, A., Mendonça, E. L., Kagan, M., Koenig, J., Krastel, H., Kroes, H. Y., Saggar, A., Sawyer, T., Schittkowski, M., Świetliński, J., Thompson, D., … Russell‐Eggitt, I. (2008). Ophthalmological aspects of Pierson syndrome. American Journal of Ophthalmology, 146(4), 602–611. 10.1016/j.ajo.2008.05.039 18672223

[mgg31704-bib-0003] Choi, H. J., Lee, B. H., Kang, J. H., Jeong, H. J., Moon, K. C., Ha, I. S., Yu, Y. S., Matejas, V., Zenker, M., Choi, Y., & Cheong, H. I. (2008). Variable phenotype of Pierson syndrome. Pediatric Nephrology (Berlin, Germany), 23(6), 995–1000. 10.1007/s00467-008-0748-7 18278520

[mgg31704-bib-0004] Cil, O., Besbas, N., Duzova, A., Topaloglu, R., Peco‐Antic, A., Korkmaz, E., & Ozaltin, F. (2015). Genetic abnormalities and prognosis in patients with congenital and infantile nephrotic syndrome. Pediatric Nephrology (Berlin, Germany), 30(8), 1279–1287. 10.1007/s00467-015-3058-x 25720465

[mgg31704-bib-0005] Cil, O., & Perwad, F. (2018). Monogenic causes of proteinuria in children. Frontiers in Medicine (Lausanne), 5, 55. 10.3389/fmed.2018.00055 PMC5858124 29594119

[mgg31704-bib-0006] Goldschmidt, D., Manoonpong, P., & Dasgupta, S. (2017). A neurocomputational model of goal‐directed navigation in insect‐inspired artificial agents. Front Neurorobot, 11, 20. 10.3389/fnbot.2017.00020 28446872 PMC5388780

[mgg31704-bib-0007] Houdayer, C., Dehainault, C., Mattler, C., Michaux, D., Caux‐Moncoutier, V., Pagès‐Berhouet, S., d'Enghien, C. D., Laugé, A., Castera, L., Gauthier‐Villars, M., & Stoppa‐Lyonnet, D. (2008). Evaluation of in silico splice tools for decision‐making in molecular diagnosis. Human Mutation, 29(7), 975–982. 10.1002/humu.20765 18449911

[mgg31704-bib-0008] Maselli, R. A., Ng, J. J., Anderson, J. A., Cagney, O., Arredondo, J., Williams, C., Wessel, H. B., Abdel‐Hamid, H., & Wollmann, R. L. (2009). Mutations in LAMB2 causing a severe form of synaptic congenital myasthenic syndrome. Journal of Medical Genetics, 46(3), 203–208. 10.1136/jmg.2008.063693 19251977 PMC2643050

[mgg31704-bib-0009] Matejas, V., Al‐Gazali, L., Amirlak, I., & Zenker, M. (2006). A syndrome comprising childhood‐onset glomerular kidney disease and ocular abnormalities with progressive loss of vision is caused by mutated LAMB2. Nephrology, Dialysis, Transplantation, 21(11), 3283–3286. 10.1093/ndt/gfl463 16921188

[mgg31704-bib-0010] Matejas, V., Hinkes, B., Alkandari, F., Al‐Gazali, L., Annexstad, E., Aytac, M. B., & Zenker, M. (2010). Mutations in the human laminin beta2 (LAMB2) gene and the associated phenotypic spectrum. Human Mutation, 31(9), 992–1002. 10.1002/humu.21304 20556798 PMC2978072

[mgg31704-bib-0011] Matejas, V., Muscheites, J., Wigger, M., Kreutzer, H. J., Nizze, H., & Zenker, M. (2011). Paternal isodisomy of chromosome 3 unmasked by autosomal recessive microcoria‐congenital nephrosis syndrome (Pierson syndrome) in a child with no other phenotypic abnormalities. American Journal of Medical Genetics Part A, 155A(10), 2601–2604. 10.1002/ajmg.a.34214 21910237

[mgg31704-bib-0012] Minamikawa, S., Miwa, S., Inagaki, T., Nishiyama, K., Kaito, H., Ninchoji, T., Yamamura, T., Nagano, C., Sakakibara, N., Ishimori, S., Hara, S., Yoshikawa, N., Hirano, D., Harada, R., Hamada, R., Matsunoshita, N., Nagata, M., Shima, Y., Nakanishi, K., … Nozu, K. (2020). Molecular mechanisms determining severity in patients with Pierson syndrome. Journal of Human Genetics, 65(4), 355–362. 10.1038/s10038-019-0715-0 31959872

[mgg31704-bib-0013] Nagano, C., Yamamura, T., Horinouchi, T., Aoto, Y., Ishiko, S., Sakakibara, N., Shima, Y., Nakanishi, K., Nagase, H., Iijima, K., & Nozu, K. (2020). Comprehensive genetic diagnosis of Japanese patients with severe proteinuria. Scientific Reports, 10(1), 270. 10.1038/s41598-019-57149-5 31937884 PMC6959278

[mgg31704-bib-0014] Noakes, P. G., Miner, J. H., Gautam, M., Cunningham, J. M., Sanes, J. R., & Merlie, J. P. (1995). The renal glomerulus of mice lacking s‐laminin/laminin beta 2: nephrosis despite molecular compensation by laminin beta 1. Nature Genetics, 10(4), 400–406. 10.1038/ng0895-400 7670489

[mgg31704-bib-0015] Pierson, M., Cordier, J., Hervouuet, F., & Rauber, G. (1963). An unusual congenital and familial congenital malformative combination involving the eye and kidney. Journal of Human Genetics, 12, 184–213. 14136829

[mgg31704-bib-0016] Richards, S., Aziz, N., Bale, S., Bick, D., Das, S., Gastier‐Foster, J., Grody, W. W., Hegde, M., Lyon, E., Spector, E., Voelkerding, K., & Rehm, H. L. (2015). Standards and guidelines for the interpretation of sequence variants: A joint consensus recommendation of the American College of Medical Genetics and Genomics and the Association for Molecular Pathology. Genetics in Medicine, 17(5), 405–424. 10.1038/gim.2015.30 25741868 PMC4544753

[mgg31704-bib-0017] Sawyer, T., Seaver, L., Loo, S., Matejas, V., & Zenker, M. (2010). Unique cardiovascular and central nervous system findings in an infant with Pierson (microcoria – congenital nephrosis) Syndrome. Journal of neonatal‐perinatal Medicine, 3(3), 233–236. 10.3233/NPM-2010-0121

[mgg31704-bib-0018] Schwartz, G. J., Brion, L. P., & Spitzer, A. (1987). The use of plasma creatinine concentration for estimating glomerular filtration rate in infants, children, and adolescents. Pediatric Clinics of North America, 34(3), 571–590. 10.1016/s0031-3955(16)36251-4 3588043

[mgg31704-bib-0019] Tahoun, M., Chandler, J. C., Ashton, E., Haston, S., Hannan, A., Kim, J. S., D’Arco, F., Bockenhauer, D., Anderson, G., Lin, M.‐H., Marzouk, S., Saied, M. H., Miner, J. H., Dattani, M. T., & Waters, A. M. (2020). Mutations in LAMB2 are associated with albuminuria and optic nerve hypoplasia with hypopituitarism. Journal of Clinical Endocrinology and Metabolism, 105(3), 595–599. 10.1210/clinem/dgz216 PMC7048679 31769495

[mgg31704-bib-0020] Wang, F., Zhang, Y. Q., Ding, J., & Yu, L. X. (2017). Detection of large deletions in X linked Alport syndrome using competitive multiplex fluorescence polymerase chain reaction. Beijing Da Xue Xue Bao Yi Xue Ban, 49(5), 760–767. 29045953

[mgg31704-bib-0021] Zenker, M., Aigner, T., Wendler, O., Tralau, T., Muntefering, H., Fenski, R., & Reis, A. (2004). Human laminin beta2 deficiency causes congenital nephrosis with mesangial sclerosis and distinct eye abnormalities. Human Molecular Genetics, 13(21), 2625–2632. 10.1093/hmg/ddh284 15367484

[mgg31704-bib-0022] Zhao, D., Ding, J., Wang, F., Fan, Q., Guan, N., Wang, S., & Zhang, Y. (2010). The first Chinese Pierson syndrome with novel mutations in LAMB2. Nephrology, Dialysis, Transplantation, 25(3), 776–778. 10.1093/ndt/gfp563 19861315

